# A novel combination of genomic loci in *ITGB2, COL5A1* and *VEGFA* associated with anterior cruciate ligament rupture susceptibility: insights from Australian, Polish, Swedish, and South African cohorts

**DOI:** 10.5114/biolsport.2026.152346

**Published:** 2025-07-16

**Authors:** Senanile B. Dlamini, Colleen J. Saunders, Paweł Cieszczyk, Krzysztof Ficek, Charlotte K. Häger, Eva-Lena Stattin, Kjell G. Nilsson, Nir Eynon, Julian A. Feller, Oren Tirosh, Christian D. Bope, Emile R. Chimusa, Mary-Jessica N. Laguette, Malcolm Collins, Alison V. September

**Affiliations:** 1 Division of Physiological Sciences, Department of Human Biology, University of Cape Town, Anatomy Building, Level 5, Anzio Road, Observatory, Cape Town, South Africa; 2 Health through Physical Activity Lifestyle and Sport Research Centre (HPALS), Division of Physiological Sciences, Department of Human Biology, 3^rd^ Floor, Sports Science Institute Building, Boundary Road, Newlands, 7701, South Africa; 3 Division of Emergency Medicine, Department of Family, Community and Emergency Care, University of Cape Town, F51 Old Main Building, Groote Schuur Hospital 7700, Cape Town, South Africa; 4 Faculty of Physical Education, Gdańsk University of Physical Education and Sport, Gdańsk, Poland; 5 Faculty of Physiotherapy, Jerzy Kukuczka Academy of Physical Education in Katowice, Katowice, Poland; 6 Department of Community Medicine and Rehabilitation, Umeå University, Umeå, Sweden; 7 Department of Immunology, Genetics and Pathology, Uppsala University, Uppsala, Sweden; 8 Department of Surgical and Perioperative Sciences, Umeå University, Umeå, Sweden; 9 Australian Regenerative Medicine Institute (ARMI), Faculty of Medicine, Nursing and Health Sciences, Monash University, Victoria, Australia; 10 OrthoSport Victoria Research Unit, Epworth Healthcare, Victoria, Australia; 11 School of Health and Biomedical Sciences, STEM College, RMIT University, Melbourne, Australia; 12 Department of Mathematics and Computer Science, Faculty of Sciences, University of Kinshasa, Kinshasa, Democratic Republic of Congo; 13 Division of Human Genetics, Department of Pathology, Faculty of Health Sciences, University of Cape Town, Cape Town, South Africa; 14 Centre for Bioinformatics, Department of Informatics, University of Oslo, Oslo, Norway; 15 Department of Applied Sciences, Faculty of Health and Life Sciences, Northumbria University, Newcastle-upon-Tyne and Wear, United Kingdom; 16 Institute of Infectious Disease and Molecular Medicine, Faculty of Health Sciences, University of Cape Town, Cape Town, South Africa; 17 International Federation of Sports Medicine (FIMS) Collaborative Centre of Sports Medicine

**Keywords:** Biomedical knowledge graph, Genetic association study, Cell signalling, Extracellular Matrix Organization pathway, Integrin protein complex

## Abstract

Integrin complexes facilitate cell communication, playing a role in ligament homeostasis. *ITGB2* rs2230528 (C/T) was implicated in anterior cruciate ligament rupture (ACL) risk in a South African cohort. Identifying biologically significant DNA signatures in the predisposition to ACL rupture risk remains important towards understanding mechanisms of ACL ruptures. *ITGB2* is essential for the activation of important biological pathways regulated by structural components such as collagens and biomechanical components such as vasculo-endothelial growth factors. This study tested the association of (i) *ITGB2* rs2230528 and (ii) allele-allele combinations of *ITGB2’s* network partners (*COL5A1* rs12722 C/T, *VEGFA* rs699947 C/A and *VEGFA* rs2010963 G/C) with ACL rupture risk. The genetic study was conducted in a combined cohort [n=1279: uninjured controls (CON), n=548; ACL ruptures (ACL), n=731; subgroup with non-contact mechanism of ACL ruptures (NON, n=425)] recruited from Australia, Poland, Sweden and South Africa. The combined cohort, rs2230528 TT (best fit model) was significantly over-represented in the ACL (p=8.00 × 10^-8^; OR:3.21; 95% CI:2.10–4.89, AIC=1549) and NON (p=1.59 × 10^-6^; OR:3.11; 95% CI:1.97–4.91, AIC=1191) groups compared to CON. *ITGB2* rs2230528-COL5A1 rs12722-*VEGFA* rs699947-*VEGFA* rs2010963, the C-C-A-G and C-T-C-G combinations were significantly associated with reduced ACL risk. This study provided additional evidence highlighting *ITGB2* as potentially being associated with ACL ruptures even though the gene-gene combinations had a small effect size. Integrins containing the b2 subunit together with its key extracellular matrix components (type V collagen and VEGFA) are potential therapeutic targets for ACL ruptures and potentially other connective tissue-related conditions.

## HIGHLIGHTS

– The genetic association study explored in this large cohort suggests key biological partners *ITGB2, COL5A1, VEGFA* in ACL risk susceptibility.
– The study also highlighted that these gene-gene combinations (*ITGB2* rs2230528, *COL5A1* rs12722, *VEGFA* rs699947, *VEGFA* rs2010963) have a small effect size on ACL risk.
– The findings of this study together with the *in-silico* findings suggest that the *ITGB2* rs2230528 variant and the ITGB2 protein be further explored at a functional and pharmacological level as a potential therapeutic target for ACL ruptures and potentially other connective tissue-related conditions.

## INTRODUCTION

Cell-cell and cell-extracellular matrix communication plays an integral role in the homeostatic regulation of the structural integrity of connective tissues such as ligaments and tendons [1]. The integrin family of proteins are key receptors in this process. By applying an *in-silico* approach, which included screening all human genes for potential links to chronic Achilles tendinopathy, Saunders *et al*. [2] identified the integrin family, specifically the integrin b2 (ITGB2) transmembrane receptor subunit, to be linked to biological pathways in tendinopathy [2]. The receptor subunit forms one of the components forming an integrin heterodimeric protein complex which is able to bind to specific ligands within the extracellular matrix environment and thereby facilitating cell migration and proliferation [3, 4]. Through these ligand binding combinations and cell migration stimulation, integrin complexes act as “stretch responses” facilitating cell signalling responses and they also form part of networks involved in the transduction of mechanical forces [5]. The *ITGB2* gene which encodes the transmembrane ITGB2 protein was identified as a predictor for anterior cruciate ligament (ACL) ruptures using whole exome sequencing in twin siblings who had sustained a non-contact ACL injury [6]. In a study aimed to identify mechanoresponsive genes related to extracellular matrix (ECM) and adhesion in human periodontal ligament cells, the *ITGB2* gene was also found to be differentially expressed within the extracellular matrix of the ligament cells [7].

ACL ruptures are caused by multiple intrinsic and extrinsic factors, and their heritability has been proposed to be as high as 80% [8]. Our previous study (Dlamini *et al.* [9]), modified a semantic biomedical knowledge graph model developed by Saunders *et al*. [2] to identify new potential risk genes for Achilles tendinopathy and ACL ruptures [9]. Through the integration of the biomedical graph and whole exome sequencing data, *ITGB2* was selected for investigation. The TT genotype and the T allele of the *ITGB2* rs2230528 C/T variant were associated with an increased risk for non-contact ACL rupture [9]. As a receptor, integrins containing the b2 subunit bind to a number of proteins as a signal conductor in order to relay specific communication in connective tissues. This allows the protein to play a crucial role in many network pathways essential for the maintenance of a healthy ACL and surrounding environment. Network partners include type V collagen, vital for collagen fibril formation, and the rs12722 C/T variant within the *COL5A1* gene, which encodes the a1(V) chain, has been associated with ligament injuries in different populations [10]. The expression of *ITGB2* and *COL5A1* were down- and up-regulated, respectively, in periodontal ligament cells in response to 24 hours of cyclic stretch [7]. It is interesting to note, that the binding of ligands such as type V collagen to the integrin heterodimer complex can activate several pro-inflammatory cytokines which are also required to facilitate the promotion of angiogenesis [5]. Vascular endothelium growth factor A, encoded by *VEGFA* is fundamental in the angiogenesis process, binds to its receptor kinase domain and together with the appropriate profile of signalling molecules will promote new blood vessel formation from existing vasculature [11]. The CC genotype of *VEGFA* rs699947 was found to have an increased risk to non-contact ACL [12] while the CC genotype for *VEGFA* rs2010963 had an increased risk for contact ACL [13]. VEGFA was also implicated in ligament biology network pathways by Feldmann *et al.* [13]. In renal cancer progression study, which used a multinetwork analysis *ITGB2* and *VEGFA* were found to be differentially expressed and implicated in the regulation of (i) leukocyte-mediated immunity, (ii) response to external stimulus and of exocytosis pathways [14].

Taken together, (i) the independent genetic associations of DNA signatures within genes *ITGB2, COL5A1* and *VEGFA* with ACL rupture susceptibility and the evidence (ii) suggesting the central role of the integrin heterodimer complex in mediating significant activation of important biological pathways involved in the homeostatic regulation of the structural (collagen fibril) and biomechanical properties (mechanotransduction) of ligament, it is reasonable to propose the collective contribution of DNA signatures in these three genes with ACL rupture susceptibility. *ITGB2, COL5A1* and *VEGFA* have complementary roles in immune regulation, extracellular matrix integrity, and angiogenesis [7, 11, 14]. Investigating the collective effect of these genes can potentially identify synergistic or additive genetic effects in ACL ruptures more accurately than single-gene analyses. This may further assist in understanding the multifactorial mechanisms of ACL ruptures and improve development of targeted therapeutic strategies.

The aim of this study was, therefore, to evaluate the *ITGB2* rs2230528 C/T variant for susceptibility to ACL rupture in three additional independent cohorts (Sweden, Poland, and Australia) and in a combined cohort analysis which included the data from the previously published South African cohort [9]. Finally, the study also aimed to explore the collective genetic contribution to ACL rupture risk susceptibility between network partners *ITGB2* rs2230528, *COL5A1* rs12722, *VEGFA* rs699947, and *VEGFA* rs2010963.

## MATERIALS AND METHODS

### Participant characteristics

This study followed a case-control genetic association design comprised of three cohorts recruited from Australia, Poland, and Sweden, and a combined cohort analysis consisting of the three cohorts and the previously published cohort from South Africa [9]. All participants were of self-reported European ancestry and completed questionnaires from their respective research centres detailing their demographic details, lifestyle habits, occupational details, sporting history (sports played, number of years, playing level, frequency), details of ACL rupture, history of other ligament or tendon rupture, and medical history. Consent was obtained in accordance with the Declaration of Helsinki. This study was approved by the Human Research Ethics Committee of the University of Cape Town, South Africa (HREC 164/2006, 622/2015, 110/2018, 269/2014, and 655/2017), the Regional Ethical Review Board in Umeå, Sweden (dnr. 2011-200-31 M), Bioethics Committee for Clinical Research, Regional Medical Chamber, Gdansk, Poland (KB-8/16) and Epworth Hospital (HREC approval: 57012) Victoria, Australia.

The cases included individuals with a clinical diagnosis of an ACL rupture (ACL-R) which was either contact or non-contact mechanism ACL injury (ACL-NON). Diagnoses was based on physical examination and confirmed by either magnetic resonance imaging or arthroscopy. Control participants (CON) included individuals who participated in regular sporting activities, similar sports to cases, had no history of an ACL rupture nor other ligament and tendon injuries, and were within a similar age category as the ACL-R group. All participants participated in regular sporting activities, primarily at a recreational level [11, 12, 13, 15, 16, 17, 18, 19, 20].

For the combined cohort analysis (n = 1279) the participants from Australia, Poland, Sweden and South Africa [9] were pooled together: controls (CON, n = 548), ACL cases (ACL-R, n = 731) and the subgroup of ACL cases with a non-contact mechanism of injury (ACL-NON, n = 425). The cohort from Australia comprised of 81 controls recruited from the Genes and Skeletal Muscle Adaptive Response to Training (Gene SMART) cohort [15], and 266 ACL rupture cases recruited between 2006 and 2018 from Epworth Richmond hospital in Melbourne, Australia. Of the 266 cases, 154 sustained their ACL rupture through non-contact mechanisms (NON subgroup) [16]. The cohort from Poland comprised 147 controls and 136 ACL cases recruited between 2009 and 2016 as previously described [17]. Male cases were recruited from Polish soccer leagues and controls from similar soccer teams while female cases were recruited from soccer teams and skiing sports. The female controls were recruited from sports clubs and wellness centres. Of the 136 cases, 54 sustained their ACL rupture through non-contact mechanisms (NON subgroup) [17]. The cohort from Sweden comprised 104 controls and 92 ACL case participants recruited between 2011 and 2013 from orthopaedic clinics in two hospitals in the cities of Umeå: Västerbotten and Luleå: Norrbotten as previously described [18]. Of the 92 cases, 76 sustained their ACL rupture through non-contact mechanisms (NON subgroup) [18]. The cohort from South Africa was previously described and genotyped [11]. In brief, the cohort consisted of 232 controls, 237 ACL cases and a subgroup of 149 non-contact mechanism, ACL rupture cases, recruited between 2006 and 2013 from sports clubs and wellness centres within Cape Town, South Africa [11].

Information on sports participation was previously published [11, 12, 13, 15, 16, 17, 18, 19, 20]. Briefly, type of sports participation for all cases and controls was categorized into contact, non-contact, non-contact non-jumping, and skiing sports. Data describing years of participation for Polish controls and all participants from Australia was not available [15, 16, 17]. Level of sport was classified as elite, national or recreational [11, 12, 13, 15, 16, 17, 18, 19, 20]. Although type and duration of sport participation was unavailable for the Australian controls, participants were moderately trained and participated in physical activities at a recreational level [15]. All male participants from the Polish cohort were matched for type of sport, level, and frequency of exposure and sports participation data for female case and control participants were comparable [17]. In the Swedish cohort, the majority of the case and control participants were involved in recreational activities [18].

### Sample collection and DNA extraction

For participants from Australia, DNA was isolated from a venous blood aliquot using a sequenced extraction technique (FlexiGene DNA Kit, Qiagen P/L) or the MagSep Blood gDNA kit with the epMotion M5073 automated pipetting system (Eppendorf). For participants from Poland, DNA was extracted from oral epithelial cells using a Gen Elute Mammalian Genomic DNA Miniprep Kit (Sigma) according to the manufacturer’s instructions. For participants from Sweden, DNA was extracted from venous blood using rapid nonenzymatic ethanol precipitation as previously described by Lahiri and Nurnberger [21] with slight modifications [22].

### Genotyping

The *ITGB2* rs2230528 (assay ID: C__25474060_10) variant was genotyped using TaqMan^TM^ assays. The standard PCR analysis was conducted using the Applied Biosystems^TM^ QuantStudio Real-Time PCR system and the Applied Biosystems^TM^ and manufacturer’s instructions were followed as previously described [9]. Negative controls (no DNA) and five repeat samples (known genotypes) were included as quality control measures for each 96-well plate. Genotypes were confirmed by two independent investigators, with an average 98.7% call rate, and laboratory work was conducted at the Health through Physical Activity Lifestyle and Sport Research Centre (HPALS) at the University of Cape Town. The *ITGB2* genotyped cohort data for South Africa was previously published. The South African cohort genotype data used for *ITGB2* (rs2230528) and the *COL5A1* (rs12722), *VEGFA* (rs699947) and *VEGFA* (rs2010963) was previously published [9, 10, 11, 12, 13, 15, 16, 17, 18, 19] and was included as part of the combined cohort analyses with permission from the senior authors of the prior studies.

### Haplotype analysis and gene-gene Combinations

Inferred haplotype analysis was conducted for *ITGB2* (rs2230528), based on the genotype data. Allele-allele combinations were explored as a proxy for potential gene-gene combinations between (*ITGB2-COL5A1-VEGFA*).

### 3D protein structure prediction and functional characterization

3D protein structure simulations were conducted to assess the effect of the rs2230528 C/T variant on the encoded ITGB2 protein. The amino acid sequences were obtained from UniProt for ITGB2 (https://www.uniprot.org/uniprot/Q14500). The tertiary structure of the *ITGB2* gene was generated using I-tasser homology webserver [23]. All simulations were conducted with the GROMACS package, version 5.6. [24–27] using Amber (AMBER99SB-ILDN) force field [28].

### Network pathway analysis

To further investigate the pathogenesis of the *ITGB2* gene, Enrichr, an enrichment analysis tool (https://maayanlab.cloud/Enrichr/) [29, 30], and GeneMANIA (https://genemania.org/) [31] were used to explore evidence for potential combinations and genetic contribution with *COL5A1* and *VEGFA* as network partners in risk assessment of ACL rupture susceptibility.

### Statistical analysis

Power calculations were calculated using QUANTO v1.2.4 (http://biostats.usc.edu/software). For the cohorts from Australia and Poland, assuming minor allele frequencies between 0.2 and 0.5, a sample size of 118 cases and greater would detect an OR 1.8 at a power of 80% and an alpha value of 0.05. For the cohort from Sweden, assuming minor allele frequencies between 0.2 and 0.5, a sample size of 85 cases would detect an allelic odds ratio (OR) 2.0, at a power of 80% and an alpha value of 0.05. The statistical program R (R Development Core Team, 2010) was used [32]. Participant descriptive statistics were compared between the mean characteristics of the CON and ACL groups using a one-way analysis of variance. The R packages genetics [33, 34] and SNPassoc [35] were used to analyse differences in genotype and allele frequencies between groups, and to calculate Hardy-Weinberg equilibrium (HWE) probabilities. HWE was tested in the control group to highlight potential genotyping errors, population substructure, and non-random sampling. In addition, five repeat samples (known genotypes) and a no DNA control sample were included as quality control measures for each 96-well plate and genotypes were confirmed by two independent investigators. The combined cohort was adjusted for country of recruitment and body mass index (BMI). Country-specific analyses were adjusted for age, height, and sex (Australia), age (Poland) or sex and BMI (Sweden). For the combined cohort, chi-square tests were used to compare genotype frequency distributions. Sex is a known intrinsic risk factor for ACL rupture susceptibility, and analyses were therefore stratified by sex [11]. All genetic models were investigated, and Aikake Information Criterion (AIC) was used to identify the best-fit model [36]. Haplotypes were inferred using the R package haplo.stats. [34]. The most common allele combination was automatically used as a reference. Statistical significance was accepted when p < 0.05, and the false discovery rate (FDR) procedure was used to adjust for multiple comparisons using the method applied for multiple testing under dependency [37]. For all the associations which included the genotype, haplotype and gene-gene combinations, analyses were conducted with and without the genotypes from participants from South Africa.

## RESULTS

### Participant characteristics

The combined CON and ACL-R, as well as the CON and ACL-NON participants were matched for sex and height (Table S1). Although the combined CON and ACL-R groups were matched for age, the combined CON (30.1 ± 12.1 years, n = 541) group was significantly older than the combined ACL-NON (28.1 ± 10.7 years, n = 418, p < 0.001) subgroup. The CON group had a significantly lower body mass (74.1 ± 13.5 kg · m^-2^, n = 536) than the ACL-R (77.7 ± 15.2 kg · m^-2^, n = 689, p < 0.001) group and ACL-NON (78.0 ± 15.3 kg · m^-2^, n = 405, p < 0.001) subgroup. Likewise, the CON group had a significantly lower BMI (23.8 ± 3.4 kg · m^-2^, n = 536) than the ACL-R group (24.8 ± 4.4 kg · m^-2^, n = 681, p = 0.001) and ACL-NON subgroup (24.7 ± 4.4 kg · m^-2^, n = 403, p = 0.005). There were no significant *ITGB2* rs2230528 genotype effects on age, sex and height in the combined cohort (Table S2 and previously published South African cohort [9]). Significant genotype effects were however noted between the C/C and C/T genotype for body mass (p = 0.009). The age, sex, height, weight, BMI as well as genotype effects of the individual Australian, Polish, Swedish and South African cohorts CON and ACL-R groups, as well as the ACLNON subgroup are summarised in Table S1, S2 or previously published for the South African cohorts [9].

### ITGB2 Genotype and Allele frequencies

**FIG. 1:**
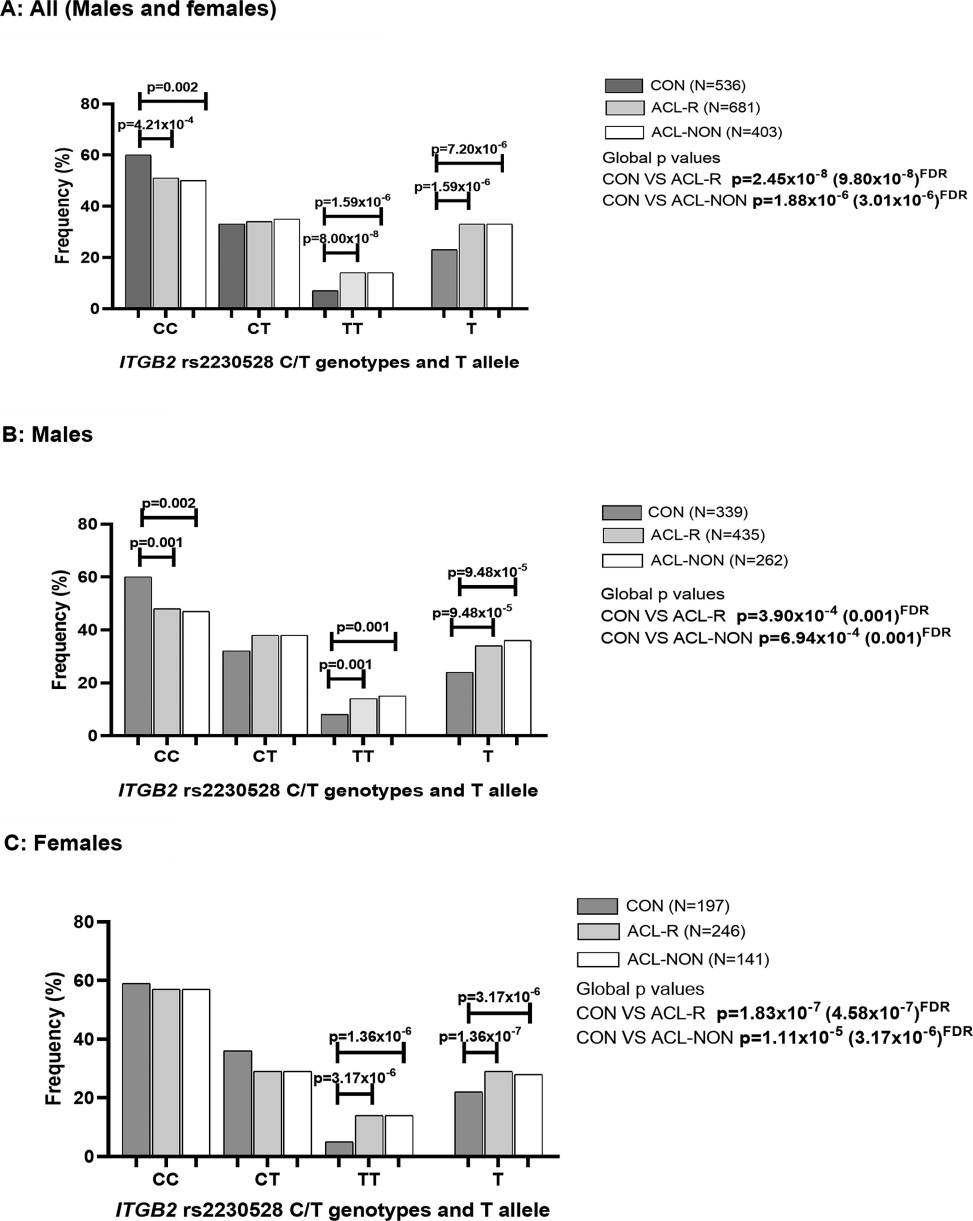
Genotype and minor allele frequency distribution for the *ITGB2* rs2230528 (C/T) polymorphisms in the combined, control (CON) group (dark grey bars), combined anterior cruciate ligament rupture (ACL-R) group (light grey bars), and non-contact mechanism ACL ruptures (ACL-NON) group (white bars) for (A) all participants, (B) males and (C) females.

In the combined cohort where the best fit model was the recessive model (TT vs CC + CT), the rs2230528 TT genotype was significantly over-represented in the ACL-R (15%, n = 681, p = 8.00 × 10^-8 FDR^, OR:3.21; 95% CI:2.10–4.89, AIC = 1549) group and ACL-NON (15%, n = 403, p = 1.59 × 10^-6 FDR^, OR:3.11; 95% CI:1.97–4.91, AIC = 1191) subgroup compared to CON group (6%, n = 536) (Figure 1A). The results of the dominant (CC vs TT + CT) and overdominant (CT vs CC + CC) models are also included in Figure 1A. The T allele was significantly over-represented in the ACL-R (33%, p = 1.59 × 10^-6 FDR^, OR:1.58; 95% CI:1.31–1.90) group and ACL-NON (33%, p = 7.20 × 10^-6 FDR^, OR:1.61 95% CI:1.31–1.99) subgroup compared to CON group (23%) (Figure 1A). Deviations from HWE were noted for both the ACL-R (p < 0.001) group and the ACL-NON (p < 0.001) subgroup.

When only male participants of the combined cohort were compared, where the best fit model was the dominant model (CC vs TT + CT), the CC genotype was significantly over-represented in the CON group (59%, n = 339) compared to ACL-R (47%, n = 435, p = 0.001 ^FDR^, OR:1.65; 95% CI:1.23–2.21, AIC = 1038) group and ACL-NON (45%, n = 262, p = 0.002 ^FDR^, OR:1.71; 95% CI:1.23–2.38, AIC = 803.0) subgroup (Figure 1B). The results of the dominant (CC vs TT + CT) and over-dominant (CT vs CC + CC) models are also included in Figure 1B. The T allele was significantly over-represented in the ACL-R (34%, p = 9.48 × 10^-5 FDR^, OR:1.63; 95% CI:1.29–2.05) group and ACL-NON (36%, p = 9.48 × 10^-5 FDR^, OR:1.72 95% CI:1.33–2.23) subgroup compared to the CON group (24%) (Figure 1B). Deviations from HWE were noted for both the ACL-R (p < 0.001) group and the ACL-NON (p < 0.001) subgroup.

**TABLE 1:** Genotype and minor allele frequency distributions, and p-values for Hardy-Weinberg exact test for *ITGB2* rs2230528 C/T in all participants (males and females), males and females for participants in the combined cohorts (Australia, Poland, Sweden, and South Africa) for the control (CON) group, anterior cruciate ligament ruptures (ACL-R) group and non-contact mechanism anterior cruciate ligament ruptures (ACL-NON) subgroup.

		CON % (n)	ACL-R % (n)	p-values^a^	AIC	ACL-NON % (n)	p-values^b^	AIC
Combined	**Males + Females**								
	n	536	681			403		
	CC	60 (320)	50 (341)	**2.45 × 10−8 (9.80 × 10−8)** **D = 3.68 × 10−4 (4.21 × 10−4**)	1569	50 (199)	**1.88 × 10−6 (3.01 × 10−6)** **D = 0.002 (0.002**)	1206
	CT	34 (181)	35 (237)	O = 0.997	1582	35 (142)	O = 0.982	1216
	TT	6 (35)	15 (103)	**R = 1.00 × 10−8 (8.00 × 10−8)**	**1549**	15 (62)	**R = 6.32 × 10−7 (1.59 × 10−6)**	**1191**
	T allele	23 (251)	33 (443)	**7.97 × 10−7 (1.59 × 10−6**)		33 (266)	**5.40 × 10−6 (7.20 × 10−6**)	
	HWE	0.097	< 0.001			< 0.001		

	**Males**								
	n	339	435			262		
	CC	59 (201)	47 (202)	**3.90 × 10−4 (0.001)** **D = 7.42 × 10−4 (0.001)**	**1038**	45 (118)	**6.94 × 10−4 (0.001)** **D = 0.001 (0.002**)	**803.0**
	CT	33 (111)	38 (167)	O = 0.160	1047	39 (101)	O = 0.230	811.6
	TT	8 (27)	15 (66)	**R = 0.001 (0.001)**	1039	16 (43)	**R = 0.001 (0.001)**	803.0
	T allele	24 (165)	34 (299)	**2.08 × 10−5 (9.48 × 10−5**)		36 (187)	**2.37 × 10−5 (9.48 × 10−5)**	
	HWE	0.084	< 0.001			< 0.001		

	**Females**								
	n	197	246			141		
	CC	60 (119)	56 (139)	**1.83 × 10−7 (4.58 × 10−7)** D = 0.052	437.7	57 (81)	**1.11 × 10−5 (1.39 × 10−5)** D = 0.125	300.7
	CT	36 (70)	29 (70)	O = 0.088	438.6	29 (41)	O = 0.126	300.7
	TT	5 (8)	15 (37)	**R = 2.71 × 10−8 (1.36 × 10−7)**	**410.6**	14 (19)	**R = 1.90 × 10−6 (3.17 × 10−6)**	**280.3**
	T allele	22 (86)	29 (144)	**0.014 (0.014)**		28 (79)	0.070	
	HWE	0.573	< 0.001			< 0.001		

Genotype and allele frequencies are expressed as a percentage with the number of participants (n) in parentheses. CON vs. ACL-Ra (adjusted P-values for country of recruitment and BMI). CON vs. ACL-NON b (adjusted P-values for country of recruitment and BMI). P-values in bold typeset indicate significance (P < 0.05). P-values corrected for multiple testing (FDR) are in parenthesis. D indicates the dominant model (CC vs TT + CT); O indicates the over-dominant model (CT vs CC + TT) and R indicates the recessive model (TT vs CC + CT). AIC indicates Akaike information criterion. AIC in bold typeset is best fit model. *ITGB2* significant p-values remained significance after FDR correction.

When females were analysed, the distribution patterns were similar to the males. The best fit model was the recessive model (TT vs CC + CT) in which the TT genotype was significantly over-represented in the ACL-R (15%, n = 246, p = 1.36 × 10^-7 FDR^, OR:8.62; 95% CI:3.68–20.18, AIC = 410.6) group and ACLNON (14%, n = 141, p = 3.17 × 10^-6 FDR^, OR:9.01; 95% CI:3.51–23.13, AIC = 280.3) subgroup compared to the CON group (4%, n = 197) (Figure 1C). The results of the dominant (CC vs TT + CT) and over-dominant (CT vs CC + CC) models are also included in Figure 1C. The T allele was significantly under-represented in the CON group (22%) compared to the ACL-R (29%, p = 0.014 ^FDR^, OR:1.48; 95% CI:1.08–2.04) group but not in the ACL-NON (28%, p = 0.070) subgroup (Figure 1C). Deviations from HWE were noted for both the ACL-R (p < 0.001) group and the ACL-NON (p < 0.001) subgroup.

**FIG. 2:**
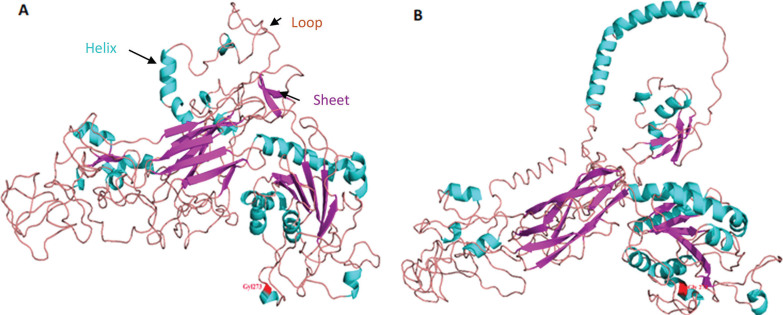
3D protein structure of the synonymous mutation the ITGB2 comparing the ancestral allele Gly1273 (red) (Figure 2A) to mutated allele Gly1273 (red) for the rs2230528 C/T SNP (Figure 2B).

**FIG. 3:**
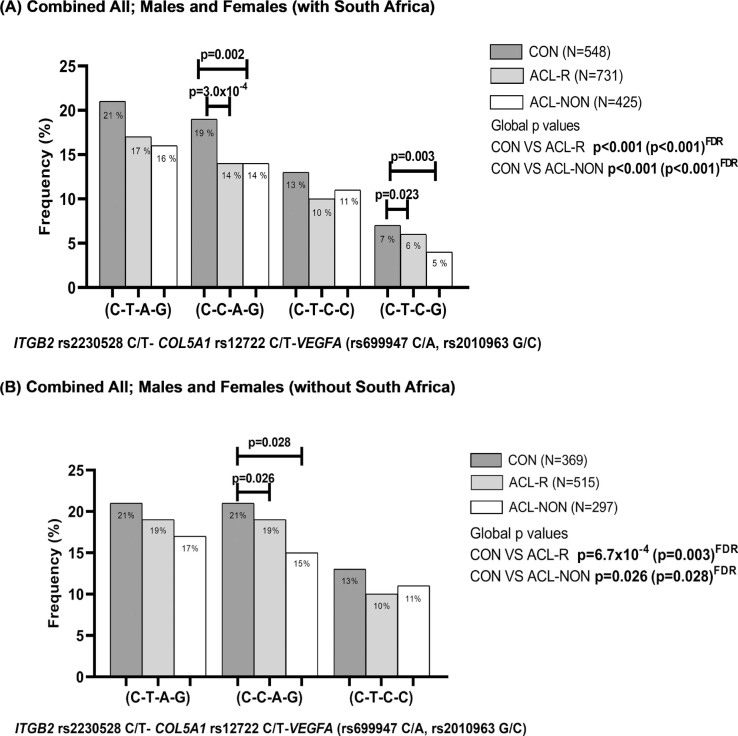
Distribution of inferred allele-allele combinations in the combined cohorts A; Australia, Poland, Sweden, and South Africa and B; Australia, Poland and Sweden constructed for *ITGB2* (rs2230528 C/T), *COL5A1* (rs12722 C/T) and *VEGFA* (rs699947 C/A, rs2010963 G/C) for ALL; Males and Females. CON: Control group (black bars), ACL-R: anterior cruciate ligament rupture group (grey bars), ACL-NON: subgroup of participants with a non-contact mechanism of injury (white bars). C-T-A-G allele combination was the most frequent combination and was selected as the reference. The number of participants (n) in each group is in parentheses. Statistically significant differences in frequency between groups are indicated, with p-values adjusted for BMI and country of recruitment. P-values in bold typeset indicate significance (p<0.05). FDR: false discovery rate.

For the independent cohorts, no significant associations were noted in Poland (CON VS ACL-R p = 0.174; CON VS ACL-NON p = 0.176) and Sweden (CON VS ACL-R p = 0.100; CON VS ACL-NON p = 0.160) (Table S4) when all participants (males and females) were analysed. However, for the Australian cohort (males and females), where the best fit model was the dominant model (CC vs TT + CT), the CC genotype was significantly over-represented in CON group (63%, n = 80) compared to the ACL-R group (55%, n = 266, p = 0.022, OR:2.19; 95% CI:1.17–4.07, AIC = 260.6) and ACLNON (53%, n = 154, p = 0.018 ^FDR^, OR:2.76; 95% CI:1.35–5.68, AIC = 191.6) subgroup. The results of the dominant (CC vs TT + CT) and over-dominant (CT vs CC + CC) models are also included in Table S4. No deviations from HWE were noted for any of the groups.

Since there were no significant differences in the *ITGB2* genotype and allele distributions between groups within the individual Polish and Swedish cohorts, and there was a strong association in the South African cohort, the combined cohort was re-analysed excluding the South African cohort. No significant associations were noted when the South African participants were excluded from the combined cohort analysis for (i) all participants (males and females), (ii) males only and (iii) females only (Table S5).

### 3D protein structure analysis

We conducted a simulation of 3D protein structure superpositions of *ITGB2* rs2230528 comparing the ancestral C allele (ancestral) to the structure produced from the alternate T allele (Figure 2) Although the polymorphism involves a synonymous amino acid (Gly) substitution at position 1273 (highlighted in red in Figure 2), the protein structure undergoes a significant transformation. Notably, the structure produced from the T allele in Figure 2B is more open with a stretched conformation when compared to the structure produced from the ancestral C allele in Figure 2A.

### Network Pathway analysis

The Enrichr tool also showed combinations between *ITGB2* and *COL5A1* through the extracellular matrix organization pathway, as well as between *ITGB2* and *VEGFA* through functional pathways involved in cell and extracellular matrix signalling. The GeneMANIA tool mined existing knowledge and showed different combinations of *ITGB2, COL5A1* and *VEGFA* interactions (physical, genetic and predicted interactions, shared protein domains, co-expression and co-localisation) and the pathways they are involved in. The *ITGB2* and *COL5A1* combination is involved in endoderm formation and cell substrate adhesion while *ITGB2* and *VEGFA* combination in cell chemotaxis and leukocyte migration functional pathways. The details on the interactions are illustrated in Figure S1 and Table S3.

### ITGB2, COL5A1 and VEGFA Allele Combinations

There were significant differences in the frequency distribution of the inferred allele combinations constructed from *ITGB2* rs22030528 (C/T), *COL5A1* rs12722 (C/T), *VEGFA* rs699947 (C/A) and *VEGFA* rs2010963 (G/C) when the combined CON group was compared to the ACL-R group or the ACL-NON sub-group (Figure 3). Specifically, the C-C-A-G combination was under-represented in the ACL-R (14%, p = 3.0 × 10^-4 FDR^, OR:0.93; 95% CI:0.61–1.41) group and ACLNON (14%, p = 0.002 ^FDR^; OR:0.96; 95% CI:0.61–1.51) subgroup compared to the CON group (19%) (Figure 3A). The C-T-C-G combination was also under-represented in the ACL-R (6%, p = 0.023 ^FDR^, OR:1.08; 95% CI:0.75–1.57) and ACL-NON (4%, p = 0.003, OR:0.55; 95% CI:0.28–1.06) subgroup compared to the CON group (7%) (Figure 3A). Similarly, significant differences in the frequency distribution of the inferred allele combinations were also noted for the combined cohort when the South African genotype frequencies were excluded, with the C-C-A-G combination was under-represented in the ACL-R (16%, p = 0.026 ^FDR^, OR:1.09; 95% CI:0.65–1.60) and ACL-NON (15%, p = 0.028 ^FDR^, OR:1.11; 95% CI:0.87–1.65) subgroup compared to the CON group (19%) (Figure 3B).

In the combined male participants, which included the South African cohort, the C-C-A-G combination, was also under-represented in the ACL-R (13%, p = 0.003 ^FDR^, OR:0.80; 95% CI:0.46–1.39) and ACL-NON (13%, p = 0.004 ^FDR^, OR:0.69; 95% CI:0.35–1.36) subgroup compared to the CON group (19%) (Figure S2A). Similarly, when the South African participants were excluded from the combined analyses for males only, the C-C-A-G combination, remained under-represented in the ACL-R (14%, p = 0.020 ^FDR^, OR:0.73; 95% CI:0.40–1.36) and ACL-NON (20%, p = 0.004 ^FDR^, OR:0.60; 95% CI:0.31–1.27) subgroup compared to the CON group (21%) (Figure S2B).

Moreover, when only the females were compared in the combined cohort (including the South African genotypes), the C-C-A-G combination, was under-represented in the ACL-R (16%, p = 0.014 ^FDR^, OR:0.94; 95% CI:0.56–1.69) and ACL-NON (16%, p = 0.044 ^FDR^, OR:1.03; 95% CI:0.56–1.90) subgroup compared to the CON group (18%) (Figure S2C). The C-T-C-G combination was also under-represented in the ACL-R (7%, p = 0.012 ^FDR^, OR:0.68; 95% CI:0.33–1.41) and ACL-NON (4%, p = 0.006 ^FDR^, OR:0.37; 95% CI:0.13–1.03) subgroup compared to the CON group (9%) (Figure S2C). While in the combined female only analyses, where the South African genotypes were excluded, the C-T-C-G combination was under-represented in the ACL-R (8%, p = 0.039 ^FDR^, OR:0.85;95% CI:0.34–2.07) and ACL-NON (4%, p = 0.024 ^FDR^, OR:0.70; 95% CI:0.23–2.18) subgroup compared to the CON group (9%) (Figure S2D).

In the independent cohorts, no significant differences in the frequency distribution of the inferred allele combinations were noted for Australia (CON VS ACL-R p = 0.223, CON VS NON p = 0.334) and Poland (CON VS ACL-R p = 0.553, CON VS ACL-NON p = 0.879) (Figure S2E,F). However, in the cohort from Sweden, the C-C-A-G combination, was under-represented in the ACL-R (4%, p = 2.0 × 10^-4 FDR^; OR:0.80; 95% CI:0.21–2.13) and ACL-NON (1%, p = 3.0 × 10^-4 FDR^, OR:0.03; 95% CI:0.00–0.65) subgroup compared to the CON group (16%) (Figure S2G). In the South African cohort, the C-C-A-G combination, was also under-represented in the ACL-R (11%, p = 2.0 × 10^-4 FDR^, OR:0.70; 95% CI:0.33–1.50) and ACL-NON (10%, p = 0.001 ^FDR^, OR:1.27; 95% CI:0.56–2.70) subgroup compared to the CON group (19%) (Figure S2H).

## DISCUSSION

Anterior cruciate ligament ruptures are considered multifactorial and both intrinsic and extrinsic factors have been implicated in the susceptibility of these injuries [38]. Genetic factors have recently received much interest with several genes and susceptibility loci being associated with predisposition to these injuries [7]. In this study, the gene-gene combinations between *ITGB2-COL5A1-VEGFA* highlighted an increased ACL rupture risk (contact and non-contact) for the C-C-A-G and C-T-C-G allele combination as noted in the combined cohort (including and excluding South Africa). These findings are highlighting potential significant biological interactions between *ITGB2-COL5A1-VEGFA* which should be considered when aiming to optimise ligament healing and improve adaptation to mechanical stress and ultimately reduce injury susceptibility [5, 13, 39]. This study investigated the *ITGB2* rs2230528 C/T variant in three independent cohorts (Sweden, Poland, and Australia) together with a previously investigated South African cohort [9] and its shared genetic contributions with network partners *COL5A1* and *VEGFA* with risk to ACL rupture. The rs2230528 C/T polymorphism involves a synonymous substitution, however, the 3D protein modelling predicts that the C > T substitution has the potential to alter the helical, sheet and loop structures of the resultant 3D protein. The bioinformatic analysis together with the gene-gene combinations reinforced the previous evidence which suggested the direct combination between *ITGB2-COL5A1* and an indirect combination between *ITGB2-COL5A1-VEGFA* in promoting inside to outside signalling or the reverse when required to regulate ECM adaption and healing [7, 14].

More specifically, when all participants (males and females) were evaluated in the combined cohort, individuals homozygous for the major allele (CC genotypes) were associated with a 2-fold reduced ACL rupture risk (contact and non-contact), while Individuals homozygous for the minor allele (TT genotypes) were associated with a 3-fold increased ACL rupture risk (contact and non-contact). The minor T allele was likewise associated with a 2-fold increased risk for sustaining an ACL injury. Similar findings were noted when male and female participants were analysed separately. When South Africa was removed from the combined cohort analyses, no significant associations were noted. For the genotype analysis, it does seem that the South African genetic profile for *ITGB2* may be driving the association noted in the combined cohort. Of the independent cohort analysis, a significant association was only noted in Australia where the CC genotype presented with a 2-fold decreased risk for contact and 3-fold decreased risk for non-contact ACL ruptures while the CT genotype, presented 2-fold increased risk for contact and 3-fold increased risk for non-contact ACL ruptures. Previous data on South African participants also identified the CC genotype to be associated with a 3-fold decreased risk for developing a contact and noncontact ACL rupture while the TT genotype showed a 7-fold increased risk for developing a contact and non-contact ACL rupture [9].

It is the gene-gene combinations that displayed the most interesting findings supporting the biological hypothesis that *ITGB2* through its ligand interactions within the ECM, potentially plays an important role in directing the signalling between various intracellular and extracellular components of the cell. Exploring this hypothesis, allele combinations between *ITGB2* rs2230528 C/T-*COL5A1* rs12722 C/T-*VEGFA* (rs699947 C/A, rs2010963 G/C) in the combined cohort (with the inclusion of South Africa) highlighted a significant association of C-C-A-G with a 1-fold reduced ACL risk (contact and non-contact). The C-T-C-G combination also showed a 1-fold reduced ACL risk (contact and non-contact) and was however, not observed when South Africa was excluded in the analyses. This combination is therefore specific to the South African cohort. Looking at the independent populations and the allele combinations, we also noted some haplotypes which are rare and absent in others. It is therefore important that large cohorts are evaluated to identify the potential common biological variants for further investigation at the functional level. It is interesting to observe that C-C-A-G remained associated with reduced ACL risk when South Africa was excluded in the combined cohort analyses. Similar findings were noted when only male or only female participants were compared in a combined cohort (with the inclusion of South Africa). It was previously proposed that ITGB2 plays an important role in influencing the biomechanical properties of the knee joint [5, 7]. Taken altogether, we hypothesise that nonsynonymous change in the *ITGB2* gene, which potentially alters the *ITGB2* protein folding, may impact the communication between ECM proteins. This disruption of the inside and outside signalling within ligaments may compromise the biomechanical properties of the knee joint.

Integrins play a significant role in mediating cell-matrix combinations and thereby influence the complex mechanotransduction of cells. More specifically, integrins, including integrins containing the β2 subunit play significant roles in various signalling pathways such as cell migration, angiogenesis, proliferation, apoptosis, and cellular differentiation [6]. The *ITGB2* gene has mostly been studied in leukocyte adhesion deficiency syndromes which are primary immunodeficiency disorders caused by defects in adhesion molecules to leukocytes and thereby disrupting the healing process of tissues. Adhesion molecules are surface bound glycoprotein molecules expressed on leukocytes and/or endothelial cells and mediate the combination between cells, or between cells and the ECM [40]. In our previous study we used Fathmm, a method that predicts functional effects of various changes within coding and non-coding regions of a gene [41] and we found the rs2230528 variant to be deleterious. Furthermore, the 3D protein dynamic simulation suggests that even though the mutation is synonymous, there is a recognisable alteration of the helical and pleated sheet arrangement of the *ITGB2* protein structure. This alteration can result in changes in the dynamic flexibility of the protein which may impact the conformation of the protein, its stability and naturally the complex binding combinations with ECM network pathway partners [42]. We hypothesised that the variant combination may affect the three-dimensional conformation of the respective proteins and thereby influence the dynamics of protein-protein binding, unwinding and dissociation. It is reasonable to glean that such structural perturbations, and in particular in pathways focused on regulating ECM homeostasis could alter the collagen fibril architecture and impact fibril stability and even delay the timely resolution of tissue repair. A less organised collagen network maybe more vulnerable to breakdown or be invaded by blood vessels and immune cells leading to the disruption of an optimal healing response, repair and recovery. We therefore hypothesised that the flexibility of the three-dimensional ITGB2 protein as influenced by this C > T substitution could potentially trigger how it engages with key sets of ECM protein partners and thereby influences the time-sensitive molecular pathways required for optimal regulation of connective tissue homeostasis.

Although there were novel associations in the study, there were some limitations. The population sizes for the individual cohorts were a limitation. The cohort from Australia did not have female controls, and it is therefore important to explore the impact of this specific variant in Australian females. We also noted the importance of improving the recruitment of participants who are matched for confounders such as the age of injury, sporting exposure, and level of sports participation. There were deviations from Hardy Weinberg noted which could be as a result of population stratification. The South African Caucasian population, largely descended from the Dutch, German, French Huguenot and British ancestry. These populations share substantial genetic ancestry with Northern and Western European populations [43]. Similarly, the Australian population of European descent predominantly originated from the British and Irish [43]. As a result, both populations exhibit genetic profiles reflective of Northern European ancestry, which emphasises their similarity in studies of genetic association and disease susceptibility [43]. Relatively recent migration histories show that these populations have experienced limited admixture and retain high levels of genetic homogeneity characteristic of many European-descended groups [44]. This genetic homogeneity across European populations has been well-documented and supports the transferability of genetic findings between European-ancestry cohorts [45]. It is therefore interesting to note that we found independent associations for *ITGB2* rs2230528 C/T in both cohorts. We did not observe this in the cohorts from Poland and Sweden. These two populations have very distinct genetic ancestry to Australia and South Africa. We do recognise that our samples have a selection bias as our cases were selectively recruited using specific inclusion criteria and similarly, our controls do not represent the general population and were also highly selected. We also acknowledge that although population of origin was included as a covariate, formal correction for population structure was not performed in this study. Therefore, the deviations for HWE in some variants may reflect unaccounted stratification, and caution is warranted when interpreting these associations.

## CONCLUSIONS

The genetic association study conducted in this combined cohort analyses identified the collective contribution of key biological partners (*ITGB2, COL5A1,* and *VEGFA)* to ACL rupture susceptibility. It was noted that these effects sizes were small. The bioinformatic analyses further supported the hypothesis that these genes playing roles in ACL homeostasis through ECM remodelling and mechanotransduction, collectively explain the variability in ACL risk susceptibility. Together, these genetic and *in-silico* findings highlight the potential of *ITGB2* as a candidate for further functional and pharmacological investigation in the context of ACL injuries and other connective tissue-related disorders.

## Supplementary Material

A novel combination of genomic loci in *ITGB2, COL5A1* and *VEGFA* associated with anterior cruciate ligament rupture susceptibility: insights from Australian, Polish, Swedish, and South African cohorts

## References

[1] Humphrey JD, Dufresne ER, Schwartz MA. Mechanotransduction and extracellular matrix homeostasis. Nat Rev Mol Cell Biol. 2014 Dec; 15(12):802–12. doi: 10.1038/nrm3896. Epub 2014 Oct 22. PMID: 25355505; PMCID: . 25355505 PMC4513363

[2] Saunders CJ Dashti MJS, Gamieldien J et al. Semantic interrogation of a multi knowledge domain ontological model of tendinopathy identifies four strong candidate risk genes. Sci. Rep 2016, 25. doi: 10.1038/srep19820. 26804977 PMC4726433

[3] Anton ES, Kreidberg JA, Rakic P. Distinct functions of α3 and α(v) integrin receptors in neuronal migration and laminar organization of the cerebral cortex. Neuron 1999, 22:277–289. 10069334 10.1016/s0896-6273(00)81089-2

[4] Nikonenko I, Toni N, Moosmayer M, Shigeri Y, Muller D, Sargent Jones L. Integrins are involved in synaptogenesis, cell spreading, and adhesion in the postnatal brain. Brain Res. Dev. Brain Res 2003, 140:185–194. 12586424 10.1016/s0165-3806(02)00590-4

[5] Tan SM. The leucocyte beta2 (CD18) integrins: the structure, functional regulation and signalling properties. Biosci. Rep 2012, 32(3):241–269. 22458844 10.1042/BSR20110101

[6] Caso E, Maestro A, Sabiers CC et al. Whole-exome sequencing analysis in twin sibling males with an anterior cruciate ligament rupture. Injury 2016, 47(3):S41–S50. doi:10.1016/S0020-1383(16)30605-2. 27692106

[7] Ma J, Zhao D, Wu Y et al. Cyclic stretch induced gene expression of extracellular matrix and adhesion molecules in human periodontal ligament cells. Arch. Oral Biol 2015, 60(3):447–455. 25541636 10.1016/j.archoralbio.2014.11.019

[8] Rahim M, Gibbon A, Collins M, September AV. Chapter fifteen— genetics of musculoskeletal soft tissue injuries: current status, challenges, and future directions. In: Barh D, Ahmetov I eds., Sports, Exercise, and Nutritional Genomics. Cambridge, MA: Academic Press; 2019:317–339.

[9] Dlamini SB, Saunders CJ, Laguette MN, Gibbon A, Gamieldien J, Collins M, September AV. Application of an in silico approach identifies a genetic locus within ITGB2, and its combinations with HSPG2 and FGF9, to be associated with anterior cruciate ligament rupture risk. Eur J Sport Sci 2023, 23:1–11. doi: 10.1080/17461391.2023.2171906. 36680346

[10] Alvarez-Romero J, Laguette MN, Seale K et al. Genetic variants within the COL5A1 gene are associated with ligament injuries in physically active populations from Australia, South Africa, and Japan. Eur J Sport Sci 2023, 23(2):284–293. doi: 10.1080/17461391.2021.2011426. Epub 2021 Dec 30. PMID: 34821541. 34821541

[11] Mannion S, Mtintsilana A, Posthumus M et al. Genes encoding proteoglycans are associated with the risk of anterior cruciate ligament ruptures. Br. J. Sports Med 2014, 48(22):1640–1646. 24552666 10.1136/bjsports-2013-093201

[12] Rahim M, Gibbon A, Hobbs H, et al. The association of genes involved in the angiogenesis-associated signaling pathway with risk of anterior cruciate ligament rupture. J Orthop Res 2014, 32:1612–1618. 25111568 10.1002/jor.22705

[13] Feldmann DC, Rahim M, Suijkerbuijk MAM, Laguette MN, Cieszczyk P, Ficek K et al. Investigation of multiple populations highlight VEGFA polymorphisms to modulate anterior cruciate ligament injury. J Orthop Res 2021, 18. doi: 10.1002/jor.25192. Epub ahead of print. PMID: 34664319. 34664319

[14] Wang A, Chen M, Wang H, Huang J, Bao Y, Gan X, Liu B, Lu X, Wang L. Cell Adhesion-Related Molecules Play a Key Role in Renal Cancer Progression by Multinetwork Analysis. Biomed Res Int 2019, 16:2325765. doi: 10.1155/2019/2325765. PMID: 31950034; PMCID: . 31950034 PMC6948336

[15] Yan X, Eynon N, Papadimitriou ID et al. The gene SMART study: method, study design, and preliminary findings. BMC Genomics 2017, 18:821. 29143594 10.1186/s12864-017-4186-4 PMC5688409

[16] Suijkerbuijk MAM, Ponzetti M, Rahim M et al. Functional polymorphisms within the inflammatory pathway regulate expression of extracellular matrix components in a genetic risk dependent model for anterior cruciate ligament injuries. J Sci Med Sport 2019, 22:1219–1225. 31395468 10.1016/j.jsams.2019.07.012

[17] Lulińska-Kuklik E, Leźnicka K, Humińska-Lisowska K et al. The VEGFA gene and anterior cruciate ligament rupture risk in the Caucasian population. Biol Sport 2019, 36:3–8. 30899133 10.5114/biolsport.2018.78902 PMC6413576

[18] Flynn RK, Pedersen CL, Birmingham TB, Kirkley A, Jackowski D, Fowler PJ. The familial predisposition toward tearing the anterior cruciate ligament: a case control study. Am J Sports Med. 2005, 33:23–28. 15610995 10.1177/0363546504265678

[19] Voisin S, Cieszczyk P, Pushkarev VP et al. EPAS1 gene variants are associated with sprint/power athletic performance in two cohorts of European athletes. BMC Genomics 2014, 15:382. 24884370 10.1186/1471-2164-15-382 PMC4035083

[20] Tengman E, Brax Olofsson L, Nilsson KG, Tegner Y, Lundgren L, Häger CK. Anterior cruciate ligament injury after more than 20 years: I. Physical activity level and knee function. Scand J Med Sci Sports. 2014 Dec; 24(6):e491–500. doi: 10.1111/sms.12212. Epub 2014 Mar 27. PMID: 24673102. 24673102

[21] Lahiri DK, Nurnberger JI Jr. A rapid non-enzymatic method for the preparation of HMW DNA from blood for RFLP studies. Nucleic Acids Res. 1991 Oct 11; 19(19):5444. doi: 10.1093/nar/19.19.5444. PMID: 1681511; PMCID: . 1681511 PMC328920

[22] Mokone GG, Gajjar M, September AV, Schwellnus MP, Greenberg J, Noakes TD, Collins M. The guanine-thymine dinucleotide repeat polymorphism within the tenascin-C gene is associated with achilles tendon injuries. Am J Sports Med. 2005 Jul; 33(7):1016–21. doi: 10.1177/0363546504271986. PMID: 15983124. 15983124

[23] Zhang Y. I-TASSER server for protein 3D structure prediction. BMC bioinformatics 2008, 9:40. 10.1186/1471-2105-9-40 PMID: 18215316. 18215316 PMC2245901

[24] Berendsen HJC, van der Spoel D, van Drunen R. GROMACS: A messagepassing parallel molecular dynamics implementation. Comp Phys Comm 1995, 91:43–56.

[25] Lindahl E, Hess B, van der Spoel D. GROMACS 3.0: A package for molecular simulation and trajectory analysis. J Mol Mod 2001, 7:306–17.

[26] Van Der Spoel D, Lindahl E, Hess B, Groenhof G, Mark AE, Berendsen HJ. GROMACS: fast, flexible, and free. J Comput Chem 2005, 26(16):1701–18. 10.1002/jcc.20291 PMID: 16211538. 16211538

[27] Pronk S, Pa´ll S, Schulz R, Larsson P, Bjelkmar P, Apostolov R et al. GROMACS 4.5: a high-throughput and highly parallel open-source molecular simulation toolkit. Bioinformatics 2013, 29(7):845–54. 10.1093/bioinformatics/btt055 PMID: 23407358. 23407358 PMC3605599

[28] Lindorff-Larsen K, Piana S, Palmo K, Maragakis P, Klepeis JL, Dror RO et al. Improved side-chain torsion potentials for the Amber ff99SB protein force field. Proteins 2010, 78(8):1950–8. 10.1002/prot.22711 PMID: 20408171. 20408171 PMC2970904

[29] Kuleshov MV, Jones MR, Rouillard AD et al. Enrichr: a comprehensive gene set enrichment analysis web server 2016 update. Nucleic Acids Res. 2016 Jul 8; 44(W1):W90–7. doi: 10.1093/nar/gkw377. Epub 2016 May 3. PMID: 27141961; PMCID: . 27141961 PMC4987924

[30] Xie Z, Bailey A, Kuleshov MV, Clarke DJB et al. Gene Set Knowledge Discovery with Enrichr. Curr Protoc. 2021 Mar; 1(3):e90. doi: 10.1002/cpz1.90. PMID: 33780170; PMCID: . 33780170 PMC8152575

[31] Warde-Farley D, Donaldson SL, Comes O et al. The GeneMANIA prediction server: biological network integration for gene prioritization and predicting gene function. Nucleic Acids Res 2010, 1(38): W214–220 PubMed Abstract (PDF). 10.1093/nar/gkq537 PMC2896186 20576703

[32] R Development Core Team. R: A language and environment for statistical computing. R Foundation for Statistical Computing. (2010) Retrieved from www.r–project.org.

[33] Warnes G, Genetics: A package for population genetics. R Package (version 1.3.6) (2011). Retrieved from http://cran.r-project.org/package=genetics.

[34] González JR, Armengol L, Solé X et al. SNPassoc: An R package to perform whole genome association studies. Bioinformatics 2007, 23(5):644–645. 17267436 10.1093/bioinformatics/btm025

[35] Sinnwell J & Schaid D. Haplo.stats: A package for statistical analysis of haplotypes with traits and covariates when linkage phase is ambiguous. R package (version 1.5.4). (2011) Retrieved from http://cran.rproject.org/package=haplo.stats

[36] Burnham KP & Anderson DR. Multimodel Inference: Understanding AIC and BIC in Model Selection. Sociol Methods Res 2004, 33:261–304.

[37] Benjamini Y & Hochberg Y. Controlling the false discovery rate: a practical and powerful approach to multiple testing. J R Stat Soc B 1995, 57(1):289–300.

[38] Collins M, Posthumus M, Schwellnus MP. The COL1A1 gene and acute soft tissue ruptures. Br J Sports Med 2010, 44(14):1063–4. doi: 10.1136/bjsm.2008.056184. Epub 2009 Feb 4. PMID: 19193665. 19193665

[39] Brown KL, Seale KB, El Khoury LY, Posthumus M, Ribbans WJ, Raleigh SM, Collins M, September AV. Polymorphisms within the COL5A1 gene and regulators of the extracellular matrix modify the risk of Achilles tendon pathology in a British case-control study. J Sports Sci 2017, 35(15):1475–1483. doi: 10.1080/02640414.2016.1221524. Epub 2016 Aug 19. PMID: 27541197. 27541197

[40] Ren G, Roberts AI, Shi Y. Adhesion molecules: key players in Mesenchymal stem cell-mediated immunosuppression. Cell Adh Migr 2011, 5(1):20–2. doi: 10.4161/cam.5.1.13491. Epub 2011 Jan 1. PMID: 20935502; PMCID: . 20935502 PMC3038091

[41] Shihab HA, Gough J, Cooper DN et al. Predicting the functional, molecular, and phenotypic consequences of amino acid substitutions using hidden Markov models. Hum. Mutat 2013, 34(1):57–65. 23033316 10.1002/humu.22225 PMC3558800

[42] Kelly SM & Price NC. The use of circular dichroism in the investigation of protein structure and function. Curr Protein Pept Sci 2000, 1(4):349–84. doi: 10.2174/1389203003381315. PMID: 12369905. 12369905

[43] de Wit E, Delport W, Rugamika CE, Meintjes A et al. Genome-wide analysis of the structure of the South African Coloured Population in the Western Cape. Hum Genet. 2010 Aug; 128(2):145–53. doi: 10.1007/s00439-010-0836-1. Epub 2010 May 20. PMID: 20490549. 20490549

[44] Campbell MC, Tishkoff SA. African genetic diversity: implications for human demographic history, modern human origins, and complex disease mapping. Annu Rev Genomics Hum Genet. 2008; 9:403–33. doi: 10.1146/annurev.genom.9.081307.164258. PMID: 18593304; PMCID: . 18593304 PMC2953791

[45] Novembre J, Johnson T, Bryc K, Kutalik Z et al. Genes mirror geography within Europe. Nature. 2008 Nov 6; 456(7218):98–101. doi: 10.1038/nature07331. Epub 2008 Aug 31. Erratum in: Nature. 2008 Nov 13; 456(7219):274. PMID: 18758442; PMCID: . 18758442 PMC2735096

