# Supplementary material for: A novel combination of genomic loci in ITGB2, COL5A1 and VEGFA associated with anterior cruciate ligament rupture susceptibility: insights from Australian, Polish, Swedish, and South African cohorts
Source: Biol Sport. 2025 Jul 16;43:3–20. doi: 10.5114/biolsport.2026.152346 (PMC12884886; doi:10.5114/biolsport.2026.152346)
Supplement: A novel combination of genomic loci in ITGB2, COL5A1 and VEGFA associated with anterior cruciate ligament rupture susceptibility: insights from Australian, Polish, Swedish, and South African cohorts [file JBS-43-56335-s1.pdf]

SUPPLEMENTARY MATERIAL

TABLE S1. Descriptive characteristics for participants in the combined cohorts (Australia, Poland, Sweden, and South Africa) for the control (CON) group, anterior cruciate ligament ruptures (ACL-R) group and non-contact mechanism anterior cruciate ligament ruptures (ACL-NON) subgroup, including the individual cohorts for Australia, Poland and Sweden.

	**Combined-CON**	**Combined-ACL-R**	**p-values**	**Combined – ACL-NON**	**p-values**
n	548	731		425	
Age (years)	30.1 ± 12.1 (541)	28.0 ± 10.6 (701)	***0.002*** (0.082) [a]	28.1 ± 10.7 (418)	***0.010 (< 0.001)*** [a]
Sex (%male)	63 (548)	63 (731)	0.907	64 (425)	0.639
Height (cm)	175.7 ± 9.5 (536)	176.0 ± 9.8 (700)	0.580	176.6 ± 9.4 (413)	0.137
Body Mass (kg)	74.1 ± 13.5 (536)	77.7 ± 15.2 (689)	***< 0.001 (< 0.001)*** [a]	78.0 ± 15.3 (405)	***< 0.001 (< 0.001)*** [a]
BMI (kg · m^{-2})	23.8 ± 3.4 (536)	24.8 ± 4.4 (681)	***< 0.001 (0.001)*** [b]	24.7 ± 4.4 (403)	***< 0.001 (0.005)*** [b]
	**Australia – CON**	**Australia – ACL-R**		**Australia – ACL-NON**	
n	81	266		154	
Age (years)	31.4 ± 8.4 (80)	25.0 ± 7.6 (266)	***< 0.001 (< 0.001)*** [c]	25.5 ± 7.5 (154)	***< 0.001 (< 0.001)*** [c]
Sex (%male)	100 (81)	55 (266)	***< 0.001***	51 (154)	***< 0.001***
Height (cm)	179.4 ± 7.0 (81)	175.1 ± 9.7 (266)	***< 0.001*** (0.272) [d]	175.4 ± 9.3 (154)	***< 0.001 (0.008)*** [d]
Body Mass (kg)	81.4 ± 11.6 (81)	77.5 ± 14.3 (266)	***0.025*** (0.070) [e]	78.0 ± 14.3 (154)	0.059
BMI (kg · m^{-2})	25.3 ± 3.2 (81)	25.2 ± 3.6 (266)	0.781	25.3 ± 3.7 (154)	0.946
	**Poland – CON**	**Poland – ACL-R**		**Poland – ACL-NON**	
n	147	136		54	
Age (years)	21.0 ± 1.8 (147)	31.3 ± 9.8 (136)	***< 0.001 (< 0.001)*** [f]	30.3 ± 9.2 (54)	***< 0.001 (< 0.001)*** [f]
Sex (%male)	75 (147)	73 (136)	0.787	87 (54)	0.083
Height (cm)	178.0 ± 9.8 (147)	177.3 ± 9.7 (136)	0.580	180.2 ± 8.9 (54)	0.138
Body Mass (kg)	72.7 ± 12.0 (147)	79.0 ± 15.0 (136)	***< 0.001*** (0.111) [g]	81.4 ± 16.9 (54)	***< 0.001 (0.034)*** [g]
BMI (kg · m^{-2})	22.8 ± 2.4 (147)	25.0 ± 4.1 (131)	***< 0.001*** (0.124) [h]	25.0 ± 5.2 (54)	***< 0.001*** (0.206) [h]
	**Sweden – CON**	**Sweden – ACL-R**		**Sweden – ACL-NON**	
n	104	92		76	
Age (years)	44.4 ± 11.8 (102)	36.9 ± 13.1 (90)	***< 0.001 (0.003)*** [i]	36.2 ± 13.4 (75)	***< 0.001 (0.002)*** [i]
Sex (%male)	33 (104)	48 (92)	**0.040**	54 (76)	***0.006***
Height (cm)	171.9 ± 9.5 (98)	172.9 ± 8.7 (82)	0.480	173.6 ± 8.7 (68)	0.250
Body Mass (kg)	71.8 ± 12.9 (97)	68.5 ± 11.3 (74)	0.086	69.2 ± 11.6 (61)	0.192
BMI (kg · m^{-2})	24.4 ± 2.7 (98)	23.0 ± 2.5 (72)	***< 0.001 (0.002)*** [j]	22.9 ± 2.5 (60)	***0.001 (0.001)*** [j]

Values are expressed as mean ± standard deviation; sex is represented as a percentage. The number of participants (n) with available data for each variable is in parentheses. P-values in bold typeset indicates significance ($p < 0.05$). CON: control group, ACL; anterior cruciate ligament rupture group, NON; non-contact mechanism anterior cruciate ligament rupture subgroup. P-values are depicted as unadjusted and adjusted in parentheses. The cohort from South Africa was previously published (Dlamini et al, [1]). P-value adjustment; [a] Age and Country of recruitment, [b] Weight and country of recruitment, [c] Height, weight and sex, [d] Age, weight and sex, [e] Age, height and sex, [f] Weight, [g] Age, [h] Age, [I] BMI and sex, [j] Age and sex.

TABLE S2. Genotype effects for participants in the combined cohorts (Australia, Poland, Sweden, and South Africa) for the control (CON) group, anterior cruciate ligament ruptures (ACL-R) group and non-contact mechanism anterior cruciate ligament ruptures (ACL-NON) subgroup, including the individual cohorts for Australia, Poland, and Sweden.

	C/C	C/T	T/T	p-values
Combined cohort				
Age (years)	29.0 ± 11.3 (681)	29.0 ± 11.4 (424)	28.7 ± 11.4 (137)	0.963
Sex (% male)	61 (690)	66 (437)	66 (152)	0.144
Height (cm)	175.4 ± 9.7 (672)	176.4 ± 9.5 (423)	176.4 ± 9.6 (138)	0.190
Body mass (kg)	75.1 ± 14.4 (666)	77.9 ± 14.7 (418)	76.1 ± 14.9 (139)	***0.008 (0.009)*** [a]
Body mass index (kg/m^2)	24.3 ± 3.8 (661)	24.7 ± 4.1 (418)	23.8 ± 4.9 (138)	0.057
Australian cohort				
Age (years)	26.4 ± 8.4 (195)	26.9 ± 7.9 (127)	24.8 ± 8.3 (24)	0.501
Sex (% male)	62 (195)	71 (128)	71 (24)	0.180
Height (cm)	175.7 ± 9.8 (195)	176.7 ± 8.7 (128)	176.7 ± 8.7 (24)	0.610
Body mass (kg)	77.1 ± 13.5 (195)	80.7 ± 14.1 (128)	75.7 ± 12.8 (24)	***0.034 (0.022)*** [a]
Body mass index (kg/m^2)	24.9 ± 3.6 (195)	25.8 ± 3.3 (128)	24.2 ± 3.1 (24)	***0.034 (0.038)*** [b]
Polish cohort				
Age (years)	26.7 ± 9.4 (169)	24.6 ± 7.0 (94)	27.0 ± 8.7 (20)	0.148
Sex (% male)	72 (169)	78 (94)	75 (20)	0.559
Height (cm)	177.0 ± 10.0 (167)	179.1 ± 9.3 (94)	176.7 ± 9.1 (20)	0.217
Body mass (kg)	75.4 ± 14.9 (168)	76.2 ± 11.9 (93)	75.9 ± 13.4 (20)	0.894
Body mass index (kg/m^2)	24.0 ± 3.9 (166)	23.7 ± 2.7 (93)	23.9 ± 2.8 (19)	0.790
Swedish cohort				
Age (years)	40.6 ± 12.7 (111)	41.3 ± 13.6 (71)	40.5 ± 12.1 (10)	0.931
Sex (% male)	37 (115)	42 (71)	50 (10)	0.641
Height (cm)	171.7 ± 8.4 (104)	172.8 ± 10.0 (66)	176.2 ± 10.9 (10)	0.281
Body mass (kg)	68.5 ± 12.3 (99)	72.3 ± 11.1 (62)	77.3 ± 16.3 (10)	***0.031*** (0.105) [b]
Body mass index (kg/m^2)	23.4 ± 2.5 (98)	24.1 ± 2.9 (62)	25.1 ± 2.9 (10)	0.087

All variables except sex are expressed as mean ± standard deviation with the number of participants presented in parentheses. Sex is expressed as percentages with the number of participants written in parentheses. Age are self-reported values at the time of the first ACL rupture of the ACL-R and the NON subgroup and at the time of recruitment for the CON group. Body mass and body mass index (BMI) are self-reported values at the time of recruitment for all groups. The p-values are presented next to the variables and significance ($p < 0.05$) is indicated in bold typeset. [a] p-value adjusted for country of recruitment; [b] p-value adjusted for age and sex. The cohort from South Africa was previously published (Dlamini *et al*, 2023).

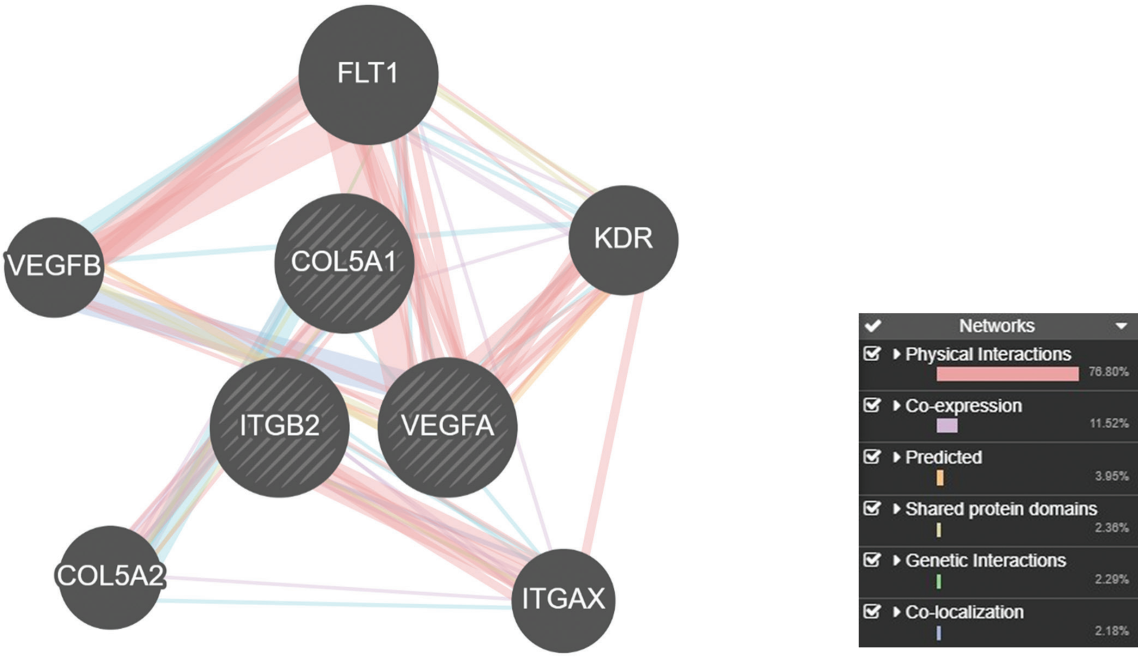

FIG. S1. ITGB2 interactions with COL5A1 and VEGFA. The type of interactions between genes indicated by different colours, are shown in the key.

TABLES3. Pathway base analysis using Enrichr and GeneMANIA, on the *ITGB2, VEGFA, COL5A1* genes.

	Interacting genes	Pathways
Enrichr (KEGG, Reactome)	ITGB2, COL5A1	Extracellular Matrix Organization
	ITGB2, VEGFA	Rheumatoid arthritis
		Interleukin-4 And Interleukin-13 signalling
		Rap1 signalling pathway
		Signalling By Interleukins
		Hemostasis
		Cytokine signalling In Immune System
GeneMANIA	ITGB2, COL5A1	Endoderm formation
		Cell substrate Adhesion
	ITGB2, VEGFA	Cell chemotaxis
		Leukocyte migration

ITGB2: Integrin subunit beta 2, COL5A1: Collagen type V alpha-1 chain: VEGFA: Vascular endothelial growth factor A Rap 1: Ras-proximate-1

TABLE S4. Genotype and minor allele frequency distributions, and p-values for Hardy-Weinberg exact test for *ITGB2* rs2230528 C/T in all participants (males and females) for participants from Australia, Poland and Sweden for the control (CON) group, anterior cruciate ligament ruptures (ACL-R) group and non-contact mechanism anterior cruciate ligament ruptures (ACL-NON) subgroup.

		CON % (n)	ACL-R % (n)	p-values[a]	AIC	ACL-NON % (n)	p-values[b]	AIC
				**Males + females**				
Australia	n	80	266			154		
	CC	63 (50)	55 (145)	**0.040 (0.040)** **D = 0.012 (0.022)**	**260.6**	52 (81)	**0.016 (0.025)** **D = 0.005 (0.018)**	**191.6**
	CT	31 (25)	38 (102)	**O = 0.018 (0.022)**	261.2	41 (63)	**O = 0.006 (0.018)**	192.1
	TT	6 (5)	7 (19)	R = 0.720	266.7	7 (10)	R = 0.790	199.5
	T allele	22 (35)	26 (140)	0.300		27 (83)	0.262	
	HWE	0.523	0.875			0.837		
				**Males + Females**				
Poland	n	147	136			54		
	CC	61 (90)	58 (79)	0.174		63 (34)	0.176	
	CT	34 (50)	32 (44)			26 (14)		
	TT	5 (7)	10 (13)			11 (6)		
	T allele	22 (64)	26 (70)	0.278		24 (26)	0.686	
	HWE	1.000	0.075			0.054		
				**Males + Females**				
Sweden	n	102	90			75		
	CC	55 (56)	61 (55)	0.100		57 (43)	0.160	
	CT	37 (38)	37 (33)			40 (30)		
	TT	8 (8)	2 (2)			3 (2)		
	T allele	26 (54)	21 (37)	0.187		23 (34)	0.456	
	HWE	0.613	0.348			0.332		

Genotype and allele frequencies are expressed as a percentage with the number of participants (n) in parentheses. CON vs. ACL-R[a] (adjusted P-values for country of recruitment and BMI). CON vs. ACL-NON [b] (adjusted P-values for country of recruitment and BMI). P-values in bold typeset indicate significance ($P < 0.05$). P-values corrected for multiple testing (FDR) are in parenthesis. D indicates the dominant model (CC vs TT + CT); O indicates the over-dominant model (CT vs CC + TT), and R indicates the recessive model (TT vs CC + CT). AIC indicates Akaike information criterion. AIC in bold typeset is best fit model. *ITGB2* significant p-values remained significance after FDR correction. In the Australian cohort ALL (males and females), the CC genotype (CC vs TT + CT) (best fit model) was over-represented in the CON group (63%) compared to the ACL-R group (55%; p = 0.022; OR:2.19; 95% CI:1.17–4.07, AIC = 260.6) and ACL-NON (52%; p = 0.018; OR:2.76; 95% CI:1.35–5.68, AIC = 191.6) subgroup. The CT (CT vs CC + TT) genotype was under-represented in the CON group (31%) compared to the ACL-R (38%; p = 0.022; OR:2.13; 95% CI:1.13–4.03, AIC = 261.2) and ACL-NON (41%; p = 0.021; OR:2.72; 95% CI:1.31–5.67, AIC = 192.1) subgroup. For the Australian cohort there were no significant differences for the recessive model (TT vs CC + CT) (TT genotype) [p = 0.720 (CON vs ACL-R), p = 0.790 (CON vs ACL-NON)] and T allele frequency [p = 0.300 (CON vs ACL-R), p = 0.262 (CON vs ACL-NON)].

TABLE S5. Genotype and minor allele frequency distributions, and p-values for Hardy-Weinberg exact test for *ITGB2* rs2230528 C/T in all participants (males and females), males and females for participants in the combined cohorts (Australia, Poland, Sweden) for the control (CON) group, anterior cruciate ligament ruptures (ACL-R) group and non-contact mechanism anterior cruciate ligament ruptures (ACL-NON) subgroup.

		CON % (n)	ACL-R % (n)	p-values[a]	ACL-NON % (n)	p-values[b]
			**Males + Females**			
	n	305	457		252	
	CC	59 (180)	57 (260)	0.881	56 (141)	0.904
	CT	35 (107)	36 (165)		37 (93)	
	TT	6 (18)	7 (32)		7 (18)	
	T allele	24 (143)	25 (229)	0.442	26 (129)	0.458
	HWE	0.537	0.472		1.000	
			**Males**			
Combined	n	207	280		148	
	CC	58 (120)	52 (146)	0.495	49 (73)	0.364
	CT	35 (74)	41 (115)		43 (64)	
	TT	7 (14)	7 (20)		8 (12)	
	T allele	25 (98)	28 (155)	0.194	30 (88)	0.100
	HWE	0.661	0.368		0.242	
			**Females**			
	n	98	177		104	
	CC	61 (60)	64 (113)	0.765	66 (69)	0.671
	CT	34 (33)	30 (53)		30 (31)	
	TT	5 (5)	6 (11)		4 (4)	
	T allele	22 (43)	21 (75)	0.917	19 (40)	0.550
	HWE	0.849	0.669		1.000	

Genotype and allele frequencies are expressed as a percentage with the number of participants (n) in parentheses. CON vs. ACL-R[a] (adjusted P-values for country of recruitment and BMI). CON vs. ACL-NON [b] (adjusted P-values for country of recruitment and BMI). P-values in bold typeset indicate significance ($P < 0.05$). P-values corrected for multiple testing (FDR) are in parenthesis. *ITGB2* significant p-values remained significance after FDR correction.

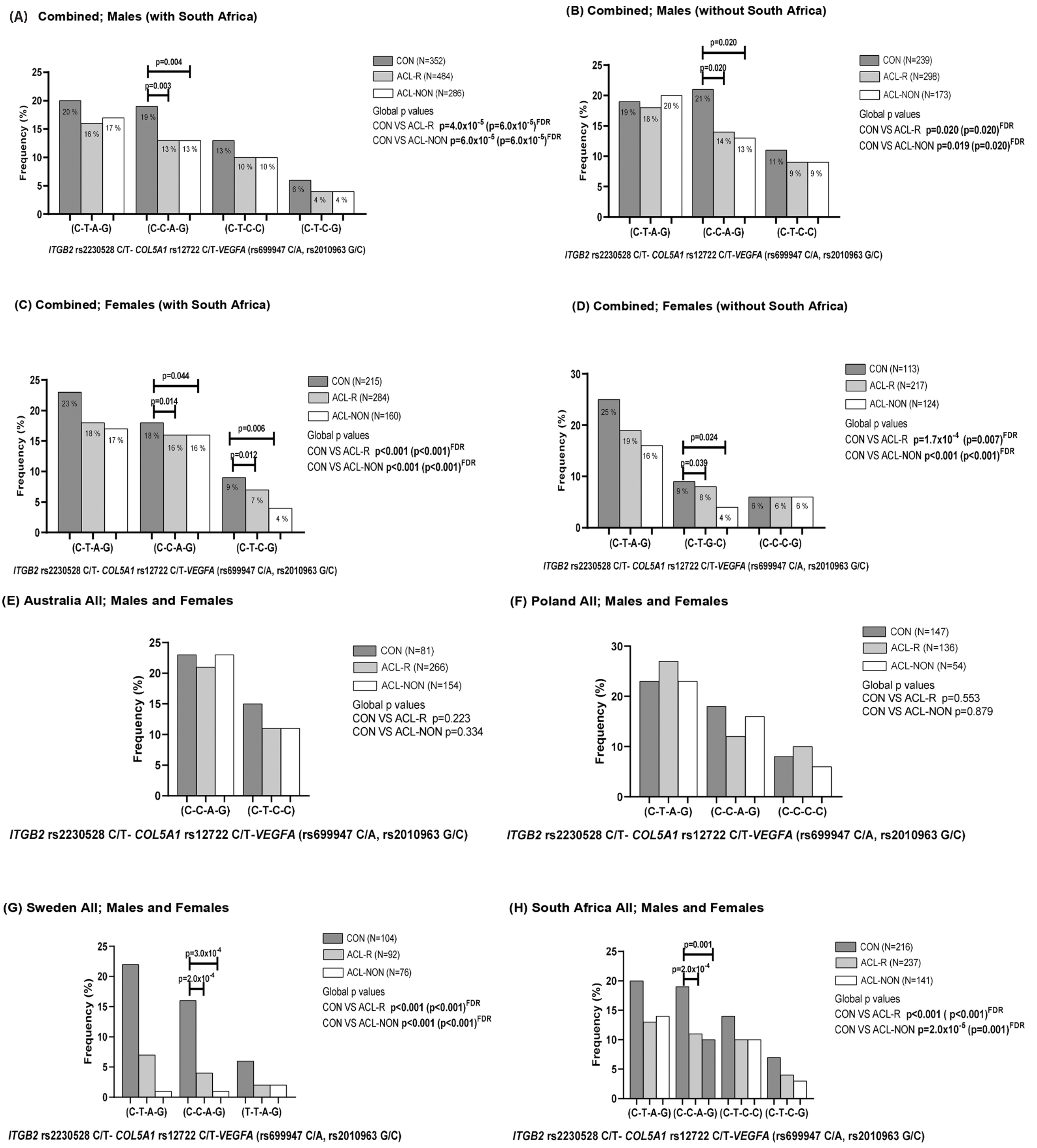

FIG. S2. Distribution of inferred allele-allele interactions in A, B, C, D; males and females of the combined cohort (including and excluding South Africa) and in E, F, G, H; individual cohorts (Australia, Poland, Sweden, and South Africa) for *ITGB2* (rs2230528 C/T)-*COL5A1* (rs12722 C/T) – *VEGFA* (rs699947 C/A, rs2010963 G/C). CON: Control group (dark grey bars), ACL-R: anterior cruciate ligament rupture group (light grey bars), ACL-NON: subgroup of participants with a non-contact mechanism of injury (white bars). C-T-A-G allele interaction was the most frequent combination and was selected as the reference. The number of participants (n) in each group is in parentheses. Statistically significant differences in frequency between groups are indicated, with p-values adjusted for BMI and country of recruitment. P-values in bold typeset indicate significance ($p < 0.05$).

REFERENCES

1. Dlamini SB, Saunders CJ, Laguette MN, Gibbon A, Gamieldien J, Collins M, September AV. Application of an in-silico approach identifies a genetic locus within ITGB2, and its interactions with HSPG2 and FGF9, to be associated with anterior cruciate ligament rupture risk. Eur J Sport Sci. 2023 Feb 23:1–11. doi: 10.1080/17461391.2023.2171906.

Articles published in the Biology of Sport are licensed under an open access Creative Commons CC BY 4.0 license.
